# Targeted Next-Generation Sequencing for the Identification of Genetic Predictors of Radiation-Induced Late Skin Toxicity in Breast Cancer Patients: A Preliminary Study

**DOI:** 10.3390/jpm11100967

**Published:** 2021-09-27

**Authors:** Sarah Cargnin, Nadia Barizzone, Chiara Basagni, Carla Pisani, Eleonora Ferrara, Laura Masini, Sandra D’Alfonso, Marco Krengli, Salvatore Terrazzino

**Affiliations:** 1 Department of Pharmaceutical Sciences, University of Piemonte Orientale, 28100 Novara, Italy; sarah.cargnin@uniupo.it; 2 Department of Health Sciences, University of Piemonte Orientale, 28100 Novara, Italy; nadia.barizzone@med.uniupo.it (N.B.); chiara.basagni@uniupo.it (C.B.); sandra.dalfonso@med.uniupo.it (S.D.); 3 Radiation Oncology, University Hospital Maggiore della Carità, 28100 Novara, Italy; carla.pisani@med.uniupo.it (C.P.); eleonora.ferrara@maggioreosp.novara.it (E.F.); laura.masini@alice.it (L.M.); marco.krengli@med.uniupo.it (M.K.); 4 Department of Translational Medicine, University of Piemonte Orientale, 28100 Novara, Italy

**Keywords:** radiotherapy, breast cancer, radiosensitivity, late skin reactions, targeted next-generation sequencing

## Abstract

Normal tissue radiosensitivity is thought to be influenced by an individual’s genetic background. However, the specific genetic variants underlying the risk of late skin reactions following radiotherapy for breast cancer remain elusive. To unravel the genetic basis for radiation-induced late skin toxicity, we carried out targeted next-generation sequencing of germline DNA samples from 48 breast cancer patients with extreme late skin toxicity phenotypes, consisting of 24 cases with grade 2–3 subcutaneous fibrosis and/or grade 2–3 telangiectasia (LENT-SOMA scales) and 24 controls with grade 0 fibrosis and grade 0 telangiectasia. In this exploratory study, a total of five single-nucleotide variants (SNVs) located in three genes (TP53, ERCC2, and LIG1) reached nominal levels of statistical significance (*p* < 0.05). In the replication study, which consisted of an additional 45 cases and 192 controls, none of the SNVs identified by targeted NGS achieved nominal replication. Nevertheless, TP53 rs1042522 (G > C, Pro72Arg) in the replication cohort had an effect (OR per C allele: 1.52, 95%CI: 0.82–2.83, *p* = 0.186) in the same direction as in the exploratory cohort (OR per C allele: 4.70, 95%CI: 1.51–14.6, *p* = 0.007) and was found be nominally associated to the risk of radiation-induced late skin toxicity in the overall combined cohort (OR per C allele: 1.79, 95%CI: 1.06–3.02, *p* = 0.028). These results raise the possibility of an association between TP53 rs1042522 and risk of radiation-induced late skin toxicity in breast cancer patients; however, large replication studies are warranted for conclusive evidence.

## 1. Introduction

Adjuvant radiotherapy (RT) after conservative surgery for early-stage breast cancer is well tolerated and effective in the majority of patients [1,2]. However, a significant proportion of long-term survivors experience late RT-related complications that may develop from six months to several years after radiation exposure [3]. Late adverse reactions to radiotherapy are generally irreversible and include a wide spectrum of normal tissue reactions, with subcutaneous fibrosis and telangiectasia being the most common late skin complications of radiotherapy for breast cancer [4]. Ionizing radiation generates reactive oxygen species that lead to localized inflammation, which ultimately evolves into a fibrotic process characterized by increased collagen deposition, poor vascularity, and scarring [5]. This leads to breast indurations and in some cases to a poor cosmetic outcome that may have an impact on patients’ quality of life [6]. Factors affecting the risk of developing late radiation-induced skin injuries include RT parameters, such as total dose given, irradiated volume, dose fractionation, and treatment duration, as well as some patient characteristics, such as their age and lifestyle [7,8].

In the last decade, there has been growing interest in the identification of genetic determinants for adverse long-term effects of radiotherapy in breast cancer patients. Regarding the development of subcutaneous fibrosis and telangiectasia following radiotherapy for breast cancer, several candidate gene association studies have focused on the polymorphic variants of genes involved in the generation of reactive oxygen species [9,10,11,12,13,14], DNA repair and damage response [10,11,12,13,14,15], and fibroblast proliferation and migration [16,17,18], as well as susceptibility genes for heritable telangiectasia-associated disorders [10,11,19,20,21,22,23]. Among the genetic variants reported to be significant in at least one study for the occurrence of radiation-induced late skin reactions of the breast are GSTA1 rs3957356 [9], GSTP1 rs1695 [13,24], SOD2 rs4880 [25], eNOS rs1799983 [9], XRCC1 rs2682585 [14], TGFβ1 rs1800469 [16,21], TP53 rs1042522 [15], and ATM rs1801516 [22]. Despite a great amount of work being carried out in the field of radiogenomics, no polymorphic gene variant has been firmly established as a risk factor for radiation-induced late skin reactions in breast cancer patients. This is at least partly because of a failure to replicate the genetic associations identified in previous studies in independent patient cohorts. In addition, only a few polymorphisms per gene are usually considered by candidate gene association studies, leading to the possibility that other overlooked genetic variants may also contribute to the risk of radiation-induced late skin toxicity in breast cancer patients.

In the present study, we hypothesized that common single-nucleotide variants (SNVs) may influence the occurrence of subcutaneous fibrosis and telangiectasia after breast irradiation for breast cancer. In order to unravel the genetic basis for radiation-induced late skin toxicity and to overcome, at least in part, the limits of previous single candidate gene association studies, we herein carried out the targeted next-generation sequencing (NGS) of a large number of candidate genes on germline DNA samples from an exploratory cohort group and, subsequently, genotyped the identified SNVs in a larger cohort of breast cancer patients who received RT after undergoing breast-conserving surgery.

## 2. Materials and Methods

### 2.1. Setting and Participants

A total of 285 female Italian patients diagnosed with breast cancer and treated with conservative surgery and adjuvant radiotherapy (RT) from 1989 to 2010 at the Department of Radiation Oncology of the University Hospital “Maggiore della Carità” in Novara, Italy, were enrolled in the present study. Radiotherapy was delivered in all patients after planning computed tomography using a 3D technique through opposed tangential 6 MV X-ray beams for a total dose of 50 Gy with daily fractions of 2 Gy, followed by 9–10 Gy electron boosts in the case of invasive lesions. At the time of patient recruitment, a peripheral blood sample was taken and stored at 4 °C until DNA extraction. The occurrence of early and late toxicity was systematically assessed by an experienced radiation oncologist at annual follow-up visits, with special attention given to telangiectasia and subcutaneous fibrosis. The mean follow-up time in the overall cohort of breast cancer patients was 10.3 years (95%CI: 9.8–10.7 years). RT-induced late adverse skin reactions were scored according to the Late Effects of Normal Tissue-Subjective Objective Management Analytical (LENT-SOMA) scale [26], which allows grading breast side effects from 1 to 4. Breast cancer patients who experienced grade ≥ 2 subcutaneous fibrosis and/or grade ≥ 2 telangiectasia (LENT-SOMA scales) were referred to as the case group and compared with control group patients with no or minimal late skin reactions of the breast (fibrosis or telangiectasia of grade 0–1).

### 2.2. DNA Extraction and Quality Control

Genomic DNA was extracted from whole blood using the QiaAmp DNA Mini Kit (Qiagen, Milan, Italy) according to the manufacturer’s protocol or with the salting-out method. The DNA concentration and purity were determined, respectively, by a Qubit 4.0 fluorometer (Thermo Fisher, Milan, Italy) and Nanodrop spectrophotometer (Thermo Fisher, Milan, Italy). DNA integrity was evaluated using the Agilent Genomic ScreenTape assay (Agilent Technologies, Milan, Italy) on the Agilent TapeStation 4200 system (Agilent Technologies, Milan, Italy).

### 2.3. NGS Library Preparation, Sequencing, and Data Analysis

Illumina sequencing libraries were prepared using the SureSelect XT HS2 DNA system (Agilent Technologies, Milan, Italy) according to the manufacturer’s instructions. The MiSeq System (Illumina, Inc., San Diego, CA, USA) was used for the NGS of a custom panel consisting of 55 genes involved in cell cycle, DNA damage repair, reactive oxygen species pathway, and encoding for profibrotic and inflammatory cytokines (Appendix A, Table A1). A secondary analysis of the sequences obtained was performed using the MiSeq Reporter software (v. 2.6.2.3, Illumina, Inc., San Diego, CA, USA) and an integrated pipeline. FastQ files were aligned to the reference genome (GRCh37/hg19) using BWA-MEM. The quality trimming of the 3′ portion of reads with low-quality scores was performed. Duplicate reads generated by PCR amplification during library preparation were removed using samtools, and we performed the local realignment of reads around indels. Variant calling was performed with the GATK software. As an acceptance quality threshold value for variant calling, we selected a Q-score of 30, corresponding to a 1:1000 error rate. Genomic and functional annotations of detected variants were made using wANNOVAR (WebANNOVAR, http://wannovar.wglab.org/, accessed on 10 April 2021).

The mean depth for the sample set was 249× (range of mean depth for the various patients: 210×–333×), with 99% of the target sequence being covered with >20 NGS reads for each individual. To obtain the genotypes of the 48 samples for the detected variants, the Variant Calling Format (VCF) files were converted into genotype calls using VCFtools. To increase the precision of this phase, we merged the 48 VCF files of the various samples into a single multi-sample VCF file. A typical VCF file only contains information about loci for which at least one of the individuals presents with a non-reference allele; therefore, we assumed patients presenting with 0 non-reference alleles for a variant to be homozygous for the reference allele for that variant.

### 2.4. Real-Time PCR

The genotyping of the SNVs selected for the replication study was conducted in the whole cohort of breast cancer patients in real-time PCR using Applied Biosystems TaqMan Pre-Designed SNP Genotyping assays (rs1042522 Assay ID: C_2403545_10; rs13181 Assay ID: C_3145033_10; rs1052555 Assay ID: C_3145034_10; rs7246696 Assay ID: C_189273455_10). Real-time PCR amplification and detection were conducted in 96-well PCR plates using a CFX Connect Real-Time PCR Detection System (Bio-Rad, Milan, Italy). Thermal cycling was initiated with a denaturation step for 10 min at 95 °C, followed by 50 cycles of 15 s at 92 °C and 90 s at 60 °C. After the PCR run was completed, allelic discrimination was analyzed using the Bio-Rad CFX Manager Software (Version 3.1, Bio-Rad). Negative and positive controls for the three genotypes were included in each real-time PCR run. For validation purposes, approximately 10% of the samples were re-genotyped, and it was found that the results were reproducible, with no discrepancies noticed in genotyping. Genotyping was performed blinded to all clinical data.

### 2.5. Statistical Analysis

Categorical variables are reported as absolute (*n*) and relative frequencies (%), while continuous variables are presented as means with standard deviation (SD). The Fishers’ exact test was first used to compare the number of reference alleles and variant alleles between cases/controls of the exploratory cohort. Univariate logistic regression analyses were then performed to calculate the odds ratios (ORs) and the 95% confidence intervals (CIs) for the association of SNVs with the risk of radiation-induced late skin toxicity, assuming an additive genetic model of inheritance (i.e., that each variant allele has an equal contribution to the outcome). To this purpose, genotypes from each SNV were coded as 0, 1, or 2 according to their number of variant alleles, with each SNV modeled as a continuous variable. Adjusted logistic regression analyses were also performed with the inclusion of confounding clinical variables (*p* < 0.05 in the respective univariate logistic regression analysis). Statistical analyses were performed using MedCalc software version 13.3.3 (MedCalc Software, Mariakerke, Belgium). The nominal significance threshold was set at *p* < 0.05, while the Bonferroni-corrected *p*-value threshold for multiple testing was set at 0.000048 (0.05/1046 SNVs) in the exploratory cohort or at 0.0125 (0.05/4 SNVs) in the replication or combined cohort. Power calculations were performed using the Quanto software (http://www.hydra.usc.edu/gxe/, accessed on 17 May 2021).

## 3. Results

### 3.1. Patients Characteristics

Detailed demographic, clinical, and RT data for the whole set of breast cancer patients after stratification according to their radiosensitive status are shown in Table 1. Overall, 69 out of the 285 breast cancer patients (24.2%) experienced moderate or severe radiation-induced late skin toxicity (grade 2–3 subcutaneous fibrosis and/or grade 2–3 telangiectasia, LENT-SOMA scales), while 216 patients had minor late skin reactions of the breast (subcutaneous fibrosis or telangiectasia of grade 0–1). The univariate logistic regression analysis revealed an association of age (OR 1.03; 95%CI, 1.00–1.06; *p* = 0.023) and BMI (OR 1.11; 95%CI, 1.03–1.19; *p* = 0.006) with a higher risk of late skin toxicity after radiation therapy (Table 1). No other clinical factor was found to be significantly different between the two groups of patients at *p* < 0.05 in the univariate analysis. The whole breast cancer population was then divided into two groups of patients. The exploratory cohort included 24 cases, randomly selected from those who experienced grade 2–3 subcutaneous fibrosis and/or grade 2–3 telangiectasia, and 24 controls with grade 0 fibrosis and grade 0 telangiectasia. The remaining breast cancer patients (*n* = 237) were included in the replication cohort, consisting of 45 cases who experienced moderate or severe subcutaneous fibrosis or telangiectasia (grades 2–3) and 192 controls with minimal or no skin fibrosis or telangiectasia (grades 0–1). There were no differences between the exploratory and replication cohorts in terms of the patients’ characteristics (Supplementary Materials Table S1), except for a higher percentage of current or former smokers in patients of the exploratory cohort (27.1% vs. 12.7%, *p* = 0.020).

### 3.2. Identification of Candidate Variants by Targeted Gene Sequencing

Targeted NGS sequencing was performed using the germline DNA of the breast cancer patients included in the exploratory cohort (*n* = 48). After quality control (QC) filtering of the raw sequencing data, 1046 SNVs were available for the comparison of allele frequency between cases (*n* = 24) and controls (*n* = 24) for the exploratory cohort. Fisher’s test revealed statistical differences at the significance level of 0.05 in the allele frequency of five SNVs located in three genes (TP53, ERCC2, and LIG1), the details of which are given in Table 2. In the univariate logistic regression analysis using an additive genetic model (Table 3), a higher risk of radiation-induced late skin toxicity was detected for the C allele of TP53 rs1042522 (OR per allele: 4.70, 95%CI: 1.51–14.6, *p* = 0.007), for the C allele of TP53 rs1642785 (OR per allele: 4.70, 95%CI: 1.51–14.6, *p* = 0.007), for the T allele of ERCC2 rs13181 (OR per allele: 4.78, 95%CI: 1.56–14.7, *p* = 0.006), for the G allele of ERCC2 rs1052555 (OR per allele: 4.75, 95%CI: 1.57–14.4, *p* = 0.006), and for the T allele of LIG1 rs7246696 (OR per allele: 2.81, 95%CI: 1.16–682, *p* = 0.022). These SNVs remained nominally associated with the risk of radiation-induced late skin toxicity after adjustment for age and BMI (Table 3). In the screened subjects, rs1042522 and rs1642785 of TP53 were found to be in complete linkage disequilibrium (LD); therefore, only one of these (rs1042522) was selected for genotyping by real-time PCR of patients included in the replication cohort. This choice was also supported by the estimation of LD between these two variants for the Italian Tuscan population (TSI, D’: 1, r^2^ = 0.98) through using the LDlink website tool (available at https://ldlink.nci.nih.gov/?tab=home, accessed on 13 April 2021). The comparison of genotypes determined by targeted NGS and real-time PCR revealed no discrepancies in the results of patients included in the exploratory study for the four SNVs selected for replication (rs1042522, rs13181, rs1052555, rs7246696).

### 3.3. Replication and Combined Cohort Analyses of SNVs Identified by Targeted NGS

In the univariate logistic regression analysis (Table 3), none of the four SNVs achieved nominal replication with an effect in the same direction as in the exploratory study. Although ERCC2 rs13181 displayed a nominal significance in the replication cohort (*p* = 0.031), the effect of the T allele of rs13181 was in the opposite direction as that shown in the exploratory study (OR per T allele: 0.60, 95%CI: 0.38–0.96). Conversely, TP53 rs1042522 was found to have an effect (OR per C allele: 1.52, 95%CI: 0.82–2.83, *p* = 0.186) in the same direction as that shown in the exploratory study and to be nominally associated with the risk of radiation-induced late skin toxicity in the overall combined cohort (OR per C allele: 1.79, 95%CI: 1.06–3.02, *p* = 0.028). However, the nominal association of TP53 rs1042522 in the overall combined cohort was lost after correction for multiple testing or after adjustment for age and BMI (OR per C allele: 1.54, 95 CI: 0.90–2.63, *p* = 0.111, Table 3).

## 4. Discussion

In the present study, we employed a targeted NGS panel of 55 candidate genes in an exploratory cohort of breast cancer patients to identify common genetic variants associated with the risk of radiation-induced late skin adverse reactions; subsequently, we attempted to replicate the nominal associations in a larger cohort of breast cancer patients. Although some SNVs were identified by targeted NGS to exhibit a nominal level of significance, none of these associations achieved replication in the validation cohort or remained significant after correction for multiple testing or adjustment for clinical covariates. Despite these negative findings, the present study provides some methodological clues for the design of future radiogenomic studies.

Our failure to replicate the findings of the exploratory cohort may be due to the use of an insufficient sample size for the replication cohort or, alternatively, to the overestimation of true genetic effects in the exploratory study, a phenomenon known as the “winner’s curse”, which represents one of the major factors contributing to the failures of many attempted replication studies [27,28]. Given the sample size of 45 cases and 192 controls used for our replication cohort and assuming a power of 80% and a nominal level of significance of 0.05, the minimal detectable odds ratio under the additive model of inheritance for the replicated SNVs (minor allele frequency (MAF) from the exploratory cohort: 0.27 to 0.42) was 2.0. Thus, the sample size of our replication cohort could be sufficient for the replication of the detected SNVs, with their ORs in the exploratory cohort being between 2.81 and 4.78. On the other hand, we cannot exclude a possible overestimation of the true genetic effects in our exploratory cohort given that breast cancer patients with late skin adverse reactions of grade 1 were included in the replication cohort but not in the exploratory cohort. Although the comparison of patient groups with the most extreme phenotypes may have been a useful approach to identify gene variants most likely associated with the endpoint of interest, this may have led to the overestimation of the true genetic effects. Despite a possible winner’s curse effect in our exploratory study, in the overall combined cohort, TP53 rs1042522 was found to be nominally associated with the risk of radiation-induced late skin toxicity using a crude (unadjusted) logistic regression analysis. Even though this result may be suggestive for a putative role of TP53 rs1042522 in the development of radiation-induced late skin toxicity, the significance of this association was lost after correction for multiple testing or adjustment for clinical covariates. Therefore, further studies using larger cohorts of breast cancer patients are required in order to clarify the effect of TP53 rs1042522 on normal tissue radiosensitivity and to take into account the potential confounding effect of clinical factors.

The TP53 gene encodes for p53, a multi-functional transcription factor involved in the induction of apoptosis, regulation of cellular proliferation, and maintenance of DNA integrity in response to genotoxic stress, including ionizing radiation [29]. The polymorphic variant rs1042522 (G > C, p.Pro72Arg) maps in the proline-rich domain of the TP53 gene, resulting in the substitution of a proline with an arginine at codon 72. The Pro/Arg allelic residues of TP53 rs1042522 have been shown to differ biochemically and biologically, leading to different levels of apoptosis [30], as well as to different effects on cell cycle progression [31] and the activation of DNA-repair genes [32]. To date, few studies have investigated the role of TP53 rs1042522 as a genetic predictor of normal tissue responses to radiation therapy in breast cancer patients; however, inconclusive results have been reported [15,33,34]. Specifically, in the study of Chang-Claude et al. [15], breast cancer patients carrying the rs1042522C allele were found to be at higher risk of telangiectasia compared to non-carriers, a finding that is in line with our results, while in the study of Tan et al. [33], no association was reported between TP53 rs1042522 and acute skin toxicity, although a trend towards decreased risk was found for carriers of the rs1042522C allele in normal-weight women. Besides TP53 rs1042522, two SNVs in ERCC2 (rs13181, rs1052555) and LIG1 rs7246696 reached a nominal level of statistical significance in our exploratory study, but these genetic variants were neither confirmed in the replication cohort nor found to be associated with the risk of radiation-induced late skin toxicity in the whole population of breast cancer patients. ERCC2 encodes for the protein excision repair cross-complementing 2 (also known as xeroderma pigmentosum group D (XPD)), which acts as an essential component of the general transcription factor IIH (TFIIH) complex [35]. In turn, TFIIH plays a crucial role in the nucleotide excision repair (NER) pathway, which is involved in the repair of a wide range of DNA lesions, including ionizing radiation-induced oxidative damage and bulky DNA adducts [36]. rs13181 and rs1052555 of ERCC2 are exonic SNVs, the former being located in exon 23 and consisting of a lysine substitution to a glutamine at position 751 (T > G, p.Lys751Gln) and the latter being a synonymous variant (G > A, p.Asp711Asp). While no data exist on the impact of rs1052555 on normal tissue radiosensitivity, the rs13181T allele (Lys 751) has been reported to lead to the sub-optimal repair of X-ray-induced DNA damage [37], but no impact of ERCC2 rs13181 has been reported in breast cancer patients in relation to the risk of radiation-induced acute skin reaction [38]. On the other hand, the LIG1 gene encodes for DNA ligase 1, which is an enzyme involved in DNA replication and base excision repair [39], and rs7246696 is an intronic variant located in the transcription factor binding site that is potentially able to influence the binding affinity of transcription factors [40].

The findings of the present study should be interpreted in light of the following limitations and considerations. First, a targeted gene NGS panel was used in the exploratory cohort; thus, we cannot exclude the possibility that some relevant genes may have been overlooked because of our limited knowledge of the genes involved in the radiation response. In addition, our exploratory cohort is limited in terms of sample size; thus, it is substantially underpowered in terms of its ability to detect associations with small genetic effect sizes. To overcome both these limitations, an approach based on whole-exome sequencing should be considered by future radiogenomic studies in order to identify the genetic determinants of normal tissue radiosensitivity in a larger exploratory cohort of cancer patients. Third, we failed to replicate the findings of the exploratory study. Again, this might reflect the low statistical power of the replication cohort, but we could not exclude the possibility that an overestimation of the true genetic effects occurred in our discovery cohort. Nevertheless, we would have expected that the effects of the SNVs selected for replication would occur in the same direction in both the exploratory and replication cohorts, while the effect of ERCC2 rs13181T allele in the two cohorts occurred in the opposite direction. Finally, the nominal association of TP53 rs1042522 in the overall combined cohort was lost after adjustment for clinical covariates. Thus, future studies using larger cohorts of breast cancer patients are warranted in order to clarify the role of TP53 rs1042522 as a genetic determinant of radiation-induced late skin toxicity. If confirmed, our results may pave the way to tailored radiotherapy strategies in breast cancer patients at higher risk of local recurrence but at lower risk of late skin toxicity, including modification of total dose and fractionation, as well as intensification of radiotherapy boost on the tumor bed. In addition, further ongoing studies will help clarify whether rare low-frequency variants with a major pathogenic role occurring in this gene panel might be involved in radiation-induced late skin toxicity.

In conclusion, the results obtained for the whole breast cancer cohort suggest a possible association between TP53 rs1042522 and the risk of radiation-induced late skin toxicity in breast cancer patients. However, the failure to confirm the association of TP53 rs1042522 within the replication cohort and the loss of its statistical significance in the overall population after correction for multiple testing or adjustment for clinical confounders indicate that we should consider this result as preliminary evidence requiring confirmation in larger cohorts of breast cancer patients. Nonetheless, our findings clearly indicate that radiogenomic studies with a two-phase design should be considered to prevent any further reports of false-positive genetic associations.

## Figures and Tables

**Table 1:** Association analysis of clinical variables with radiation-induced late adverse skin reactions in the whole population of breast cancer patients (*n* = 285).

Clinical Variable	All *n* (%)	Grade 0–1 *n* (%)	Grade ≥ 2 *n* (%)	OR (95% CI)	*p*-Value
Age, years (SD)	60.8 (10.1)	60.0 (10.0)	63.2 (10.0)	1.03 (1.00–1.06)	0.023
BMI, mean (SD)	25.0 (3.8)	24.6 (3.8)	26.2 (3.7)	1.11 (1.03–1.19)	0.006
Breast diameter, cm (SD)	12.2 (2.6)	12.0 (2.6)	12.7 (2.6)	1.10 (1.00–1.22)	0.054
Breast CTV, cc (SD)	394.2 (530.0)	380.8 (586.5)	438.3 (270.4)	1.00 (1.00–1.00)	0.489
Follow-up, years (SD)	10.3 (4.0)	10.0 (3.9)	11.0 (4.2)	1.06 (0.99–1.13)	0.071
Diabetes mellitus					
No	267 (93.7)	205 (94.9)	62 (89.9)	1 (reference)	
Yes	18 (6.3)	11 (5.1)	7 (10.1)	2.10 (0.78–5.66)	0.140
Hypertension					
No	211 (74.0)	155 (71.8)	56 (81.2)	1 (reference)	
Yes	74 (26.0)	61 (28.2)	13 (18.8)	0.60 (0.30–1.16)	0.124
Vascular disease					
No	263 (92.3)	200 (92.6)	63 (91.3)	1 (reference)	
Yes	22 (7.7)	16 (7.4)	6 (8.7)	1.19 (0.45–3.17)	0.727
Tabagism					
Never	242 (84.9)	181 (83.8)	61 (88.4)	1 (reference)	
Current or former	43 (15.1)	35 (16.2)	8 (11.6)	0.68 (0.30–1.54)	0.354
Alcohol					
No	276 (96.8)	209 (96.8)	67 (97.1)	1 (reference)	
Yes	9 (3.2)	7 (3.2)	2 (2.9)	0.89 (0.18–4.39)	0.887
Postsurgical complications					
None	239 (83.9)	176 (81.5)	63 (91.3)	1 (reference)	
Seromas and hematomas	46 (16.1)	40 (18.5)	6 (8.7)	0.42 (0.17–1.04)	0.060
Neoadjuvant CT					
No	278 (97.5)	210 (97.2)	68 (98.6)	1 (reference)	
Yes	7 (2.5)	6 (2.8)	1 (1.4)	0.51 (0.06–4.35)	0.542
Adjuvant treatments					
None	40 (14.0)	28 (13.0)	12 (17.4)	1 (reference)	
CT	61 (21.4)	48 (22.2)	13 (18.8)	0.63 (0.25–1.57)	0.324
HT	131 (46.0)	100 (46.3)	31 (44.9)	0.72 (0.33–1.59)	0.420
CT + HT	53 (18.6)	40 (18.5)	13 (18.8)	0.76 (0.30–1.90)	0.556
Radiation quality					
X-rays	263 (92.3)	199 (92.1)	64 (92.8)	1 (reference)	
γ-rays	22 (7.7)	17 (7.9)	5 (7.2)	0.91 (0.32–2.58)	0.866
Dose/fraction					
2 Gy	274 (96.1)	208 (96.3)	66 (95.7)	1 (reference)	
1,8 Gy	11 (3.9)	8 (3.7)	3 (4.3)	1.18 (0.30–4.58)	0.809
Boost dose/fraction					
3 Gy	72 (25.3)	58 (26.9)	14 (20.3)	1 (reference)	
1.5–2 Gy	189 (66.3)	139 (64.4)	50 (72.5)	1.49 (0.76–2.90)	0.241
No boost	24 (8.4)	19 (8.8)	5 (7.2)	1.09 (0.35–3.43)	0.882
Acute skin toxicity, RTOG grade					
0–1	196 (68.8)	151 (69.9)	45 (65.2)	1 (reference)	
≥2	89 (31.2)	65 (30.1)	24 (34.8)	1.24 (0.70–2.20)	0.465

BMI, body mass index; CI, confidence interval; CT, chemotherapy; CTV, clinical target volume; OR, odds ratio; HT, hormone therapy; *n*, number; RTOG, Radiation Therapy Oncology Group; SD, standard deviation.

**Table 2:** Single-nucleotide polymorphisms identified by target sequencing for association with radiation-induced late adverse skin reactions (SOMA-LENT scales) in the exploration cohort of breast cancer patients (*n* = 48).

Gene	Chr	SNP	BP	Region	Ref (Allele 1)/ Alt (Allele 2)	RAF Grade 0/ RAF Grade ≥ 2	*p*-Value *	Risk Allele
TP53	17	rs1042522	7579472	Exonic (p.Pro72Arg)	G/C	0.40/0.15	0.011	C
TP53	17	rs1642785	7579801	UTR5	G/C	0.40/0.15	0.011	C
ERCC2	19	rs13181	45854919	Exonic (p.Lys751Gln)	G/T	0.50/0.23	0.010	T
ERCC2	19	rs1052555	45855524	Exonic (p.Asp711Asp)	A/G	0.44/0.17	0.007	G
LIG1	19	rs7246696	48673212	Intronic	C/T	0.56/0.31	0.023	T

* *p*-values are from Fisher’s test. Alt, alternative allele; BP, base position based on GrCh37/hg19 assembly; Chr, chromosome; RAF, reference allele frequency; Ref, reference allele.

**Table 3:** Association analysis between SNVs identified by targeted NGS and risk of radiation-induced late adverse skin reactions in breast cancer patients.

SNP	Cohort	Genotype Count		Crude OR (95%CI)	*p*-Value	Adjusted OR * (95%CI)	*p*-Value
		Controls 11/12/22	Cases 11/12/22				
TP53 rs1042522	Exploratory	3/13/8	0/7/17	4.70 (1.51–14.6)	0.007	4.23 (1.25–14.4)	0.021
	Replication	7/74/111	2/11/32	1.52 (0.82–2-83)	0.186	1.27 (0.68–2.39)	0.456
	Combined	10/87/119	2/18/49	1.79 (1.06–3.02)	0.028	1.54 (0.90–2.63)	0.111
ERCC2 rs13181	Exploratory	5/14/5	0/11/13	4.78 (1.56–14.7)	0.006	4.14 (1.27–13.4)	0.018
	Replication	24/83/85	10/22/13	0.60 (0.38–0.96)	0.031	0.61 (0.38–0.97)	0.036
	Combined	29/97/90	10/33/26	0.90 (0.61–1.33)	0.595	0.90 (0.60–1.4)	0.598
ERCC2 rs1052555	Exploratory	4/13/7	0/8/16	4.75 (1.57–14.4)	0.006	4.09 (1.30–12.9)	0.016
	Replication	22/81/89	9/20/16	0.67 (0.42–1.07)	0.093	0.64 (0.40–1.03)	0.064
	Combined	26/94/96	9/28/32	1.02 (0.68–1.52)	0.922	0.98 (0.65–1.46)	0.916
LIG1 rs7246696	Exploratory	8/11/5	2/11/11	2.81 (1.16–6.82)	0.022	2.72 (1.09–6.80)	0.032
	Replication	27/81/84	10/17/18	0.80 (0.51–1.24)	0.316	0.81 (0.51–1.28)	0.365
	Combined	35/92/89	12/28/29	0.99 (0.68–1.45)	0.971	1.00 (0.68–1.47)	0.993

* Logistic regression analysis adjusted by age and BMI, under the additive genetic model.

## Data Availability

Data are contained within the article.

## Supplementary Materials

The following are available online at https://www.mdpi.com/article/10.3390/jpm11100967/s1, Table S1: Comparison of patients’ characteristics between the exploratory (*n* = 48) and the validation cohort (*n* = 237).

## Abbreviations

BER: base excision repair; DR: direct repair; DSB: double-strand breaks; HR: homologous recombination; MMR: mismatch repair; NER: nucleotide excision repair; NHEJ: nonhomologous end joining.

## Appendix A

**Table A1:** List of genes selected for targeted next-generation sequencing.

Pathway Function	Genes
Cell cycle	BAX, CDKN1A, CHEK1, CHEK2, ESPL1, RAD9A
DNA damage repair: BER	APEX1, APEX2, LIG3, OGG1, PNKP, XRCC1
DNA damage repair: DR	MGMT
DNA damage repair: MMR	MLH1, MSH2, MSH3, MSH6, PMS2, SSBP1
DNA damage repair: NER	ERCC1, ERCC2, ERCC3, ERCC4, ERCC5, ERCC6, ERCC8, LIG1, PCNA
DSB detection	MRE11A, NBN, RAD50
DSB repair: HR	BRCA1, BRCA2, RAD51, RAD51C, XRCC2, XRCC3
DSB repair: NHEJ	LIG4, PRKDC, XRCC4, XRCC5, XRCC6
DSB signal transduction	ATM, ATR, TP53, TP53BP1
Profibrotic and inflammatory cytokines	ACVRL1, IL12RB2, TGFB1, TNF-α, IL-6
Reactive oxygen species pathway	GSTA1, GSTP1, NOS3, SOD2
