# Supplementary material for: Targeted Next-Generation Sequencing for the Identification of Genetic Predictors of Radiation-Induced Late Skin Toxicity in Breast Cancer Patients: A Preliminary Study
Source: J Pers Med. 2021 Sep 27;11(10):967. doi: 10.3390/jpm11100967 (PMC8540941; doi:10.3390/jpm11100967)
Supplement: Supplementary file 1 [file jpm-11-00967-s001.zip › jpm-1336451-supplementary.pdf]

Supplementary Table S1. Comparison of patients'characteristics between the exploratory (n=48) and the validation cohort (n=237).

Clinical variable	Exploratory cohort<br>Mean (SD) or n (%)	Validation cohort<br>Mean (SD) or n (%)	p-value*
Age, years (SD)	59.5 (9.8)	61.1 (10.1)	0.320
BMI, mean (SD)	25.4 (3.6)	24.9 (3.9)	0.448
Breast diameter, cm (SD)	12.1 (2.6)	12.2 (2.7)	0.790
Breast CTV, cc (SD)	351.5 (215.4)	403.2 (574.7)	0.316
Follow-up, years (SD)	10.4 (3.5)	10.3 (4.1)	0.847
Diabetes mellitus			0.729
No	46 (95.8)	221 (93.2)	
Yes	2 (4.2)	16 (6.8)	
Hypertension			0.462
No	33 (68.8)	178 (75.1)	
Yes	15 (31.3)	59 (24.9)	
Vascular disease			0.903
No	45 (93.8)	218 (92.0)	
Yes	3 (6.3)	19 (18.0)	
Tabagism			0.020
Never	35 (72.9)	207 (87.3)	
Current or former	13 (27.1)	30 (12.7)	
Alcohol			0.989
No	46 (95.8)	230 (97.0)	
Yes	2 (4.2)	7 (3.0)	
Post-surgical complications			
None	39 (81.3)	200 (84.4)	0.746
Seromas and hematomas	9 (18.8)	37 (15.6)	
Neoadjuvant CT			0.743
No	47 (97.9)	231 (97.5)	
Yes	1 (2.1)	6 (2.5)	
Adjuvant treatments			0.245
None	7 (14.6)	33 (13.9)	
Chemotherapy (C)	13 (27.1)	48 (20.3)	
Hormone Therapy (HT)	16 (33.3)	115 (48.5)	
C+HT	12 (25.0)	41 (17.3)	
Radiation quality			0.903
X-rays	44 (91.7)	219 (92.4)	
γ-rays	4 (8.3)	18 (7.6)	
Dose/fraction			0.772
2 Gγ	47 (97.9)	227 (95.8)	
1,8 Gγ	1 (2.1)	10 (4.2)	
Boost dose/fraction			0.731
3 Gγ	10 (20.8)	62 (26.2)	
1.5-2 Gγ	34 (70.8)	155 (65.4)	
No boost	4 (8.3)	20 (8.4)	
Acute skin toxicity, RTOG grade			0.861
0-1	32 (66.7)	164 (69.2)	
≥ 2	16 (33.3)	73 (30.8)	

*The chi-squared test was used for assessing differences among groups in the distribution of categorical variables, while the Student's t-test or the Welch's F test was applied, respectively, for continuous variables with equal or unequal variances. BMI, body mass index; CT, chemotherapy; CTV, clinical target volume; HT, hormone therapy; n, number; RTOG, Radiation Therapy Oncology Group; SD, standard deviation.
